# 3,4-Diamino­benzonitrile

**DOI:** 10.1107/S1600536813005151

**Published:** 2013-02-28

**Authors:** David K. Geiger, Dylan E. Parsons

**Affiliations:** a Department of Chemistry, State University of New York-College at Geneseo, 1 College Circle, Geneseo, NY 14454, USA

## Abstract

The non-H atoms in the structure of the title mol­ecule, C_7_H_7_N_3_, are almost coplanar (r.m.s. deviation = 0.018 Å). The two amine groups each donate two and accept one weak N—H⋯N hydrogen bonds. N—H⋯N hydrogen bonding between the amine and nitrile groups results in chains parallel to [101] in the crystal structure. The chains are cross-linked by N—H⋯N hydrogen bonds between amine groups, giving rise to an infinite three-dimensional network.

## Related literature

For the crystal structures of related compounds, see: Czapik & Gdaniec (2010 ▶); Stålhandske (1981 ▶).
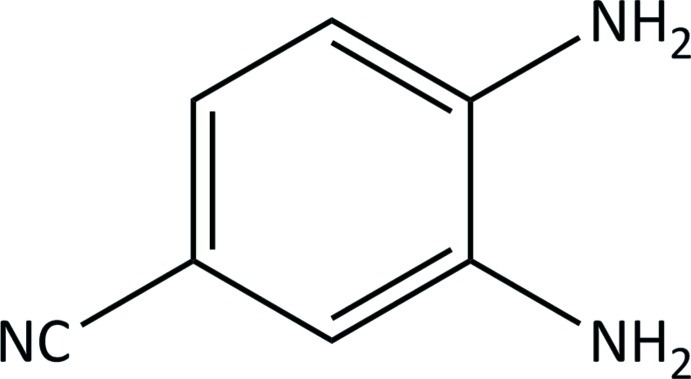


## Experimental

### 

#### Crystal data

C_7_H_7_N_3_
*M* *_r_* = 133.16
Monoclinic, 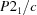

*a* = 8.858 (3) Å
*b* = 10.536 (4) Å
*c* = 8.160 (3) Å
β = 116.213 (12)°
*V* = 683.2 (4) Å^3^
*Z* = 4
Mo *K*α radiation
μ = 0.08 mm^−1^
*T* = 200 K
0.50 × 0.20 × 0.10 mm

#### Data collection

Bruker SMART X2S CCD diffractometer
Absorption correction: multi-scan (*SADABS*; Bruker, 2010 ▶) *T*_min_ = 0.85, *T*_max_ = 0.99
2149 measured reflections
1188 independent reflections
662 reflections with *I* > 2σ(*I*)
*R*_int_ = 0.039

#### Refinement

*R*[*F* ^2^ > 2σ(*F* ^2^)] = 0.052
*wR*(*F* ^2^) = 0.128
*S* = 0.96
1188 reflections
107 parameters
H atoms treated by a mixture of independent and constrained refinement
Δρ_max_ = 0.18 e Å^−3^
Δρ_min_ = −0.19 e Å^−3^

### 

Data collection: *APEX2* (Bruker, 2010 ▶); cell refinement: *SAINT* (Bruker, 2010 ▶); data reduction: *SAINT*; program(s) used to solve structure: *SHELXS97* (Sheldrick, 2008 ▶); program(s) used to refine structure: *SHELXL97* (Sheldrick, 2008 ▶); molecular graphics: *XSHELL* (Bruker, 2010 ▶) and *Mercury* (Macrae *et al.*, 2008 ▶); software used to prepare material for publication: *publCIF* (Westrip, 2010 ▶).

## Supplementary Material

Crystal structure: contains datablock(s) global, I. DOI: 10.1107/S1600536813005151/qk2053sup1.cif

Structure factors: contains datablock(s) I. DOI: 10.1107/S1600536813005151/qk2053Isup2.hkl

Supplementary material file. DOI: 10.1107/S1600536813005151/qk2053Isup3.mol

Supplementary material file. DOI: 10.1107/S1600536813005151/qk2053Isup4.cml

Additional supplementary materials:  crystallographic information; 3D view; checkCIF report

## Figures and Tables

**Table 1:** Hydrogen-bond geometry (Å, °)

*D*—H⋯*A*	*D*—H	H⋯*A*	*D*⋯*A*	*D*—H⋯*A*
N1—H1*A*⋯N2^i^	0.89 (3)	2.37 (3)	3.251 (4)	168 (3)
N1—H1*B*⋯N3^ii^	0.91 (3)	2.31 (3)	3.147 (4)	154 (3)
N2—H2*A*⋯N1^iii^	0.90 (3)	2.36 (3)	3.246 (4)	173 (2)
N2—H2*B*⋯N3^ii^	0.93 (3)	2.42 (3)	3.303 (4)	159 (3)

Symmetry codes: (i) 
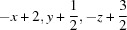
; (ii) 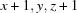; (iii) 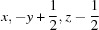.

**Fig. 1.**
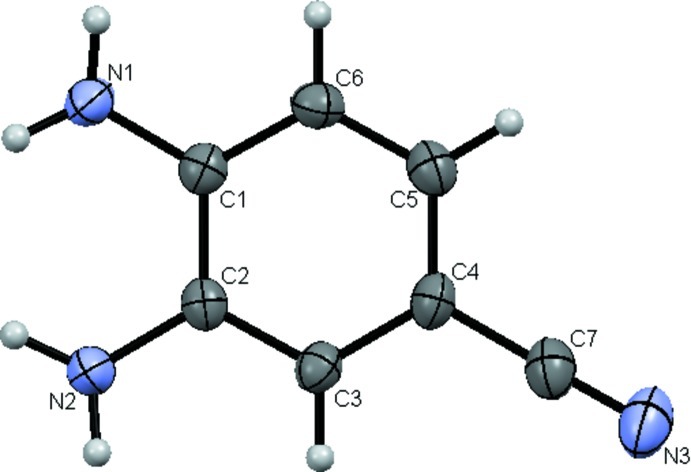
Perspective view of the title molecule. Displacement ellipsoids of the nonhydrogen atoms are drawn at the 50% probability level.

**Fig. 2.**
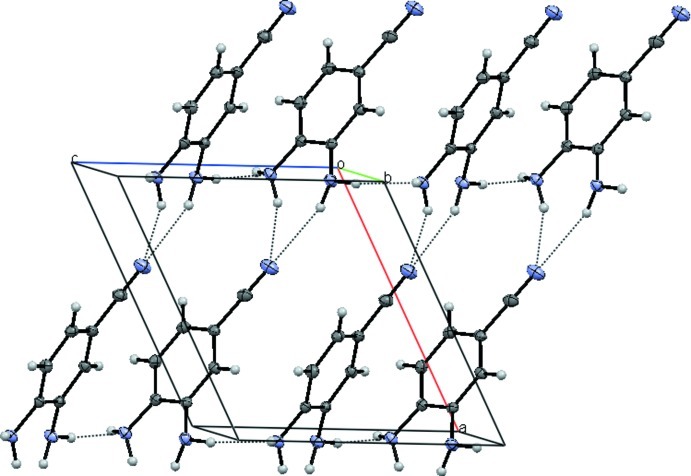
A view of the unit cell showing the chains parallel to [101] resulting from H-bonding between amine and nitrile groups and the cross-linking H-bonds between amine groups. Displacement ellipsoids of the nonhydrogen atoms are drawn at the 25% probability level.

## supplementary crystallographic information

### Comment

Single crystals of the title compound were obtained during its purification by recrystallization for use in the synthesis of an organometallic complex. Figure 1 shows a view of the title molecule with the atom numbering scheme. The non-hydrogen atoms are planar with a r. m. s. deviation of 0.018 Å. The maximum deviation is for C1, which is 0.034 (2) Å out of the plane. The benzene ring is planar with a maximum deviation of 0.008 (2) Å for C4. The amine nitrogens are decidely pyramidal with H-N-H angles of 113 (3)° and 112 (2)° for N1 and N2, respectively. N1 and N2 are 0.056 (4) and 0.073 (4) Å, respectively, below the benzene plane. All four of the hydrogen atoms of the amine groups are on the opposite side of the benzene plane from the amine nitrogen atoms. The nitrile group carbon and nitrogen atoms are 0.046 (4) and 0.086 (5) Å, respectively, out of the benzene plane and are on the opposite side as the amine nitrogens. The nitrile is linear (C4-C7-N = 179.2 (3)°).

There are two known crystalline forms of 1,2-diaminobenzene (Stålhandske, 1981; Czapik & Gdaniec, 2010). In both forms, one of the N-H bonds of each amine group is coplanar with the benzene ring and an intramolecular N-H···N interaction is exhibited. Intermolecular hydrogen bonding results in layers that are joined by additional hydrogen-bonding interactions. The two forms are isostructural in two dimensions, but differ in the stacking of the layers (Czapik & Gdaniec, 2010). Figure 2 shows the hydrogen-bonding network exhibited by the title compound. In contrast to 1,2-diaminobenzene, no intramolecular hydrogen-bonding is observed. Parallel chains of molecules with an interplanar spacing of 3.32Å are formed by hydrogen-bonds involving one hydrogen atom from each of the amines and the nitrile group on adjacent molecules. The chains run parallel to [101] and are crosslinkeded by hydrogen bonds between amine groups.

### Experimental

The compound was obtained commercially (Sigma-Aldrich). Single crystals were grown by slow evaporation of an ethanolic solution.

### Refinement

The H atoms bonded to carbon were refined using a riding model with C—H = 0.95 Å and *U*_iso_ = 1.2*U*_eq_(C). The coordinates and isotropic thermal parameters of the amine H atoms were refined freely.

### Crystal data

**Table:**

C_7_H_7_N_3_	*F*(000) = 280
*M**_r_* = 133.16	*D*_x_ = 1.294 Mg m^−^^3^
Monoclinic, *P*2_1_/*c*	Mo *K*α radiation, λ = 0.71073 Å
Hall symbol: -P 2ybc	Cell parameters from 516 reflections
*a* = 8.858 (3) Å	θ = 2.6–21.2°
*b* = 10.536 (4) Å	µ = 0.08 mm^−^^1^
*c* = 8.160 (3) Å	*T* = 200 K
β = 116.213 (12)°	Prism, colourless
*V* = 683.2 (4) Å^3^	0.50 × 0.20 × 0.10 mm
*Z* = 4	

### Data collection

**Table:**

Bruker SMART X2S CCD diffractometer	1188 independent reflections
Radiation source: fine-focus sealed tube	662 reflections with *I* > 2σ(*I*)
Graphite monochromator	*R*_int_ = 0.039
Detector resolution: 8.33 pixels mm^-1^	θ_max_ = 25.2°, θ_min_ = 3.2°
ω scans	*h* = −10→10
Absorption correction: multi-scan (*SADABS*; Bruker, 2010)	*k* = −12→11
*T*_min_ = 0.85, *T*_max_ = 0.99	*l* = −5→9
2149 measured reflections	

### Refinement

**Table:**

Refinement on *F*^2^	Primary atom site location: structure-invariant direct methods
Least-squares matrix: full	Secondary atom site location: difference Fourier map
*R*[*F*^2^ > 2σ(*F*^2^)] = 0.052	Hydrogen site location: inferred from neighbouring sites
*wR*(*F*^2^) = 0.128	H atoms treated by a mixture of independent and constrained refinement
*S* = 0.96	*w* = 1/[σ^2^(*F*_o_^2^) + (0.0526*P*)^2^] where *P* = (*F*_o_^2^ + 2*F*_c_^2^)/3
1188 reflections	(Δ/σ)_max_ < 0.001
107 parameters	Δρ_max_ = 0.18 e Å^−^^3^
0 restraints	Δρ_min_ = −0.19 e Å^−^^3^

### Special details

**Table:**

Experimental. Refinement of F^2^ against ALL reflections. The weighted *R*-factor *wR* and goodness of fit S are based on F^2^, conventional *R*-factors *R* are based on F, with F set to zero for negative F^2^. The threshold expression of F^2^ > 2sigma(F^2^) is used only for calculating *R*-factors(gt) *etc*. and is not relevant to the choice of reflections for refinement. *R*-factors based on F^2^ are statistically about twice as large as those based on F, and R– factors based on ALL data will be even larger.
Geometry. All e.s.d.'s (except the e.s.d. in the dihedral angle between two l.s. planes) are estimated using the full covariance matrix. The cell e.s.d.'s are taken into account individually in the estimation of e.s.d.'s in distances, angles and torsion angles; correlations between e.s.d.'s in cell parameters are only used when they are defined by crystal symmetry. An approximate (isotropic) treatment of cell e.s.d.'s is used for estimating e.s.d.'s involving l.s. planes.
Refinement. Refinement of *F*^2^ against ALL reflections. The weighted *R*-factor *wR* and goodness of fit *S* are based on *F*^2^, conventional *R*-factors *R* are based on *F*, with *F* set to zero for negative *F*^2^. The threshold expression of *F*^2^ > σ(*F*^2^) is used only for calculating *R*-factors(gt) *etc*. and is not relevant to the choice of reflections for refinement. *R*-factors based on *F*^2^ are statistically about twice as large as those based on *F*, and *R*- factors based on ALL data will be even larger.

### Fractional atomic coordinates and isotropic or equivalent isotropic displacement parameters (Å^2^)

**Table:**

	*x*	*y*	*z*	*U*_iso_*/*U*_eq_	
N1	1.0289 (3)	0.4587 (3)	0.7729 (3)	0.0317 (7)	
H1A	1.025 (3)	0.538 (3)	0.809 (4)	0.053 (10)*	
H1B	1.135 (4)	0.433 (3)	0.798 (4)	0.063 (11)*	
N3	0.3764 (3)	0.3161 (2)	−0.0415 (3)	0.0495 (8)	
N2	1.0370 (3)	0.2330 (2)	0.5881 (3)	0.0327 (7)	
H2A	1.044 (3)	0.180 (3)	0.507 (4)	0.035 (8)*	
H2B	1.142 (4)	0.264 (3)	0.670 (4)	0.068 (11)*	
C1	0.8972 (3)	0.4302 (2)	0.6030 (3)	0.0259 (7)	
C2	0.9008 (3)	0.3168 (2)	0.5098 (3)	0.0253 (7)	
C3	0.7650 (3)	0.2879 (2)	0.3464 (3)	0.0276 (7)	
H3	0.7664	0.2122	0.2838	0.033*	
C4	0.6251 (3)	0.3686 (3)	0.2712 (3)	0.0298 (7)	
C7	0.4877 (3)	0.3387 (3)	0.0985 (4)	0.0366 (8)	
C5	0.6212 (3)	0.4783 (3)	0.3641 (3)	0.0323 (8)	
H5	0.5262	0.5329	0.3150	0.039*	
C6	0.7562 (3)	0.5071 (3)	0.5275 (3)	0.0305 (7)	
H6	0.7525	0.5820	0.5905	0.037*	

### Atomic displacement parameters (Å^2^)

**Table:**

	*U*^11^	*U*^22^	*U*^33^	*U*^12^	*U*^13^	*U*^23^
N1	0.0325 (16)	0.0300 (17)	0.0274 (13)	−0.0019 (12)	0.0086 (13)	−0.0039 (12)
N3	0.0410 (15)	0.0481 (18)	0.0418 (15)	−0.0042 (13)	0.0022 (14)	0.0047 (13)
N2	0.0311 (14)	0.0309 (15)	0.0300 (13)	0.0049 (12)	0.0080 (12)	−0.0032 (12)
C1	0.0271 (15)	0.0256 (16)	0.0251 (14)	−0.0025 (12)	0.0115 (13)	0.0030 (12)
C2	0.0233 (14)	0.0259 (16)	0.0268 (14)	−0.0006 (12)	0.0111 (13)	0.0047 (13)
C3	0.0278 (14)	0.0264 (17)	0.0258 (14)	−0.0028 (12)	0.0092 (13)	0.0008 (12)
C4	0.0237 (14)	0.0338 (18)	0.0277 (15)	−0.0037 (13)	0.0074 (13)	0.0011 (13)
C7	0.0305 (16)	0.036 (2)	0.0387 (17)	0.0015 (13)	0.0108 (16)	0.0062 (14)
C5	0.0272 (15)	0.0324 (19)	0.0366 (16)	0.0020 (13)	0.0134 (14)	0.0036 (13)
C6	0.0345 (16)	0.0252 (16)	0.0327 (16)	0.0016 (13)	0.0156 (14)	−0.0015 (12)

### Geometric parameters (Å, º)

**Table:**

N1—C1	1.395 (3)	C2—C3	1.379 (3)
N1—H1A	0.89 (3)	C3—C4	1.401 (3)
N1—H1B	0.91 (3)	C3—H3	0.9500
N3—C7	1.157 (3)	C4—C5	1.392 (4)
N2—C2	1.401 (3)	C4—C7	1.432 (3)
N2—H2A	0.90 (3)	C5—C6	1.375 (3)
N2—H2B	0.93 (3)	C5—H5	0.9500
C1—C6	1.384 (3)	C6—H6	0.9500
C1—C2	1.424 (3)		
			
C1—N1—H1A	113.1 (17)	C2—C3—H3	119.5
C1—N1—H1B	119 (2)	C4—C3—H3	119.5
H1A—N1—H1B	113 (3)	C5—C4—C3	119.7 (2)
C2—N2—H2A	112.9 (16)	C5—C4—C7	120.2 (2)
C2—N2—H2B	119 (2)	C3—C4—C7	120.0 (2)
H2A—N2—H2B	112 (2)	N3—C7—C4	179.2 (3)
C6—C1—N1	120.7 (3)	C6—C5—C4	119.4 (2)
C6—C1—C2	118.8 (2)	C6—C5—H5	120.3
N1—C1—C2	120.4 (2)	C4—C5—H5	120.3
C3—C2—N2	120.6 (2)	C5—C6—C1	122.0 (3)
C3—C2—C1	119.1 (2)	C5—C6—H6	119.0
N2—C2—C1	120.2 (2)	C1—C6—H6	119.0
C2—C3—C4	121.0 (2)		

### Hydrogen-bond geometry (Å, º)

**Table:**

*D*—H···*A*	*D*—H	H···*A*	*D*···*A*	*D*—H···*A*
N1—H1*A*···N2^i^	0.89 (3)	2.37 (3)	3.251 (4)	168 (3)
N1—H1*B*···N3^ii^	0.91 (3)	2.31 (3)	3.147 (4)	154 (3)
N2—H2*A*···N1^iii^	0.90 (3)	2.36 (3)	3.246 (4)	173 (2)
N2—H2*B*···N3^ii^	0.93 (3)	2.42 (3)	3.303 (4)	159 (3)

Symmetry codes: (i) −*x*+2, *y*+1/2, −*z*+3/2; (ii) *x*+1, *y*, *z*+1; (iii) *x*, −*y*+1/2, *z*−1/2.
